# Supplementary material for: Assessment of agro-morphological, physiological and yield traits diversity among tropical rice
Source: PeerJ. 2021 Jul 19;9:e11752. doi: 10.7717/peerj.11752 (PMC8297474; doi:10.7717/peerj.11752)
Supplement: Supplemental Information 2 — Phenotypic variability in physiological (net photosynthesis (Pn), Stomata conductance (Cond), Transpiration rate (Tr), intrinsic Water use efficiency (iWUE), chlorophyll fluorescence (Fv/Fm), Electron transport rate (ETR), Total chlorophyll (Chl) and Carotenes (Caro)), agro-morphological traits (days to heading (DH, d), tiller number (TN, per plant), plant height (PHT, cm) and shoot dry weight (SHW, g per plant)) and yield-related traits (panicle length (PN, cm), spikelet number per panicle (SPN, no per panicle), grain weight per panicle (GW, g per panicle), spikelet fertility (SF, %), grain number per plant (GN, per plant), grain yield (GY, g per plant) and harvest index (HI, plant basis)) traits values among 74 rice genotypes under non-stress conditions. Each value is the mean of 5 replications and two years pooled data. PCA-based grouping was performed using the factor scores of PC1 and PC2. As suggested by the PCA, 74 rice genotypes were classified into four groups (Fig. 1C). Group (G) 1 and 2 included each 24 rice genotypes, group 3 included 17, and group 4 included 15 genotypes (Fig. 1C). In the following sections, we present phenotypic variability of all traits based on PCA-derived grouping. [file peerj-09-11752-s002.docx]

**Table S1:** Phenotypic variability in physiological (net photosynthesis (Pn), Stomata conductance (Cond), Transpiration rate (Tr), intrinsic Water use efficiency (iWUE), chlorophyll fluorescence (Fv/Fm), Electron transport rate (ETR), Total chlorophyll (Chl) and Carotenes (Caro)), agro-morphological traits (days to heading (DH, d), tiller number (TN, per plant), plant height (PHT, cm) and shoot dry weight (SHW, g per plant)) and yield-related traits (panicle length (PN, cm), spikelet number per panicle (SPN, no per panicle), grain weight per panicle (GW, g per panicle), spikelet fertility (SF, %), grain number per plant (GN, per plant), grain yield (GY, g per plant) and harvest index (HI, plant basis)) traits values among 74 rice genotypes under non-stress conditions. Each value is the mean of 5 replications and two years pooled data. PCA-based grouping was performed using the factor scores of PC1 and PC2. As suggested by the PCA, 74 rice genotypes were classified into four groups (Fig. 1C). Group (G) 1 and 2 included each 24 rice genotypes, group 3 included 17, and group 4 included 15 genotypes (Fig. 1C). In the following sections, we present phenotypic variability of all traits based on PCA-derived grouping.

| **Genotype** | **PCA-Group** | **Pn** | **Cond** | **Tr** | **iWUE** | **Fv'/Fm'** | **ETR** | **Chl** | **Caro** | **DH** | **TN** | **PH** | **SHW** | **PL** | **SPN** | **GW** | **SF** | **GN** | **GY** | **HI** |
| --- | --- | --- | --- | --- | --- | --- | --- | --- | --- | --- | --- | --- | --- | --- | --- | --- | --- | --- | --- | --- |
| COL XXI | G1 | 32.04 | 1.38 | 12.62 | 2.56 | 0.53 | 162.33 | 43.83 | 7.08 | 139.70 | 52.00 | 79.00 | 173.76 | 27.35 | 13.85 | 2.67 | 82.76 | 152.65 | 81.59 | 0.32 |
| CT18233 | G1 | 34.22 | 1.80 | 13.28 | 2.58 | 0.50 | 169.25 | 41.55 | 5.86 | 132.30 | 36.00 | 83.80 | 139.79 | 23.58 | 14.80 | 3.16 | 89.38 | 150.65 | 55.43 | 0.28 |
| CT18237 | G1 | 32.58 | 1.51 | 12.02 | 2.76 | 0.54 | 170.86 | 38.37 | 5.67 | 124.20 | 46.00 | 78.40 | 180.70 | 27.80 | 13.40 | 3.17 | 83.21 | 139.10 | 74.58 | 0.29 |
| CT18244 | G1 | 30.64 | 1.25 | 11.83 | 2.68 | 0.57 | 147.48 | 42.82 | 7.41 | 124.80 | 52.00 | 77.50 | 205.08 | 25.08 | 12.37 | 3.74 | 85.30 | 173.42 | 105.07 | 0.34 |
| CT18245 | G1 | 31.86 | 1.11 | 11.85 | 2.76 | 0.49 | 168.91 | 39.12 | 6.04 | 125.70 | 53.00 | 83.40 | 202.44 | 26.80 | 10.55 | 2.04 | 86.35 | 103.90 | 74.44 | 0.27 |
| CT18247 | G1 | 34.60 | 1.36 | 12.04 | 2.89 | 0.86 | 226.18 | 36.28 | 5.62 | 95.50 | 44.00 | 81.10 | 165.32 | 24.20 | 12.40 | 2.57 | 87.61 | 151.45 | 85.53 | 0.34 |
| FED 473 | G1 | 34.86 | 1.39 | 11.49 | 3.09 | 0.51 | 146.08 | 35.24 | 4.93 | 122.70 | 52.00 | 81.50 | 173.76 | 23.41 | 10.35 | 2.36 | 89.41 | 111.06 | 83.22 | 0.32 |
| FEDE 21 | G1 | 28.42 | 0.87 | 10.20 | 2.82 | 0.54 | 159.12 | 42.61 | 6.80 | 129.90 | 61.00 | 91.10 | 238.98 | 24.33 | 13.55 | 2.52 | 82.74 | 157.85 | 86.64 | 0.27 |
| HHZ 12 | G1 | 30.52 | 1.09 | 11.37 | 2.69 | 0.47 | 151.38 | 45.77 | 4.86 | 127.60 | 54.00 | 92.50 | 197.30 | 24.20 | 14.00 | 2.46 | 80.98 | 140.60 | 83.93 | 0.30 |
| IR07F287 | G1 | 29.04 | 1.01 | 11.67 | 2.64 | 0.51 | 143.44 | 46.08 | 7.14 | 92.30 | 74.00 | 72.10 | 139.37 | 21.98 | 11.35 | 1.96 | 74.15 | 148.90 | 124.41 | 0.47 |
| IR07K142 | G1 | 29.86 | 1.12 | 10.23 | 2.89 | 0.51 | 167.95 | 49.19 | 8.53 | 89.70 | 72.00 | 70.50 | 149.45 | 19.45 | 11.90 | 2.29 | 78.29 | 159.45 | 118.72 | 0.44 |
| IR08A172 | G1 | 35.60 | 1.45 | 13.23 | 2.81 | 0.53 | 138.07 | 43.26 | 6.89 | 89.60 | 66.00 | 74.20 | 117.55 | 22.30 | 12.85 | 2.79 | 81.93 | 199.42 | 107.83 | 0.48 |
| IR08N136 | G1 | 36.76 | 1.74 | 10.98 | 3.37 | 0.57 | 168.69 | 45.71 | 7.41 | 90.80 | 62.00 | 74.10 | 142.48 | 21.70 | 11.70 | 2.94 | 76.10 | 168.85 | 90.12 | 0.39 |
| IR09A130 | G1 | 32.48 | 1.16 | 12.55 | 2.62 | 0.53 | 150.17 | 38.77 | 6.23 | 97.50 | 66.00 | 75.90 | 140.49 | 21.63 | 12.00 | 2.73 | 86.52 | 182.80 | 109.39 | 0.44 |
| IR09L337 | G1 | 36.04 | 1.39 | 11.91 | 3.17 | 0.50 | 144.02 | 41.03 | 6.12 | 92.40 | 72.00 | 74.90 | 139.68 | 19.43 | 10.95 | 2.02 | 63.19 | 114.45 | 81.63 | 0.37 |
| IR10N230 | G1 | 34.98 | 2.01 | 12.23 | 2.94 | 0.50 | 181.58 | 32.83 | 5.28 | 122.30 | 44.00 | 74.20 | 154.08 | 26.03 | 12.95 | 2.70 | 74.80 | 141.25 | 59.16 | 0.28 |
| IR64-NIL | G1 | 30.16 | 1.28 | 11.15 | 2.82 | 0.53 | 150.83 | 36.76 | 5.95 | 116.30 | 71.00 | 85.60 | 228.54 | 24.30 | 10.45 | 2.55 | 78.04 | 128.65 | 106.77 | 0.32 |
| IR78221 | G1 | 35.50 | 1.67 | 13.76 | 2.57 | 0.47 | 162.65 | 45.05 | 7.04 | 123.20 | 47.00 | 79.80 | 181.50 | 26.50 | 12.85 | 2.72 | 73.00 | 135.45 | 80.23 | 0.31 |
| IR78222 | G1 | 34.50 | 1.14 | 11.73 | 2.98 | 0.50 | 139.70 | 44.86 | 6.95 | 121.90 | 53.00 | 87.80 | 215.11 | 26.50 | 13.15 | 2.62 | 73.84 | 161.70 | 68.79 | 0.24 |
| IR86174-1 | G1 | 32.14 | 1.21 | 11.10 | 2.96 | 0.51 | 177.96 | 41.95 | 5.81 | 100.40 | 53.00 | 78.60 | 153.87 | 20.85 | 21.80 | 2.78 | 84.98 | 151.10 | 88.86 | 0.37 |
| Rex | G1 | 33.42 | 1.42 | 13.00 | 2.58 | 0.53 | 153.25 | 38.92 | 6.33 | 101.40 | 17.50 | 77.30 | 106.70 | 20.95 | 12.75 | 2.75 | 85.58 | 152.75 | 48.40 | 0.31 |
| 12DS-15 | G2 | 27.60 | 1.33 | 11.69 | 2.43 | 0.53 | 149.02 | 37.20 | 5.81 | 117.80 | 45.00 | 75.70 | 147.42 | 22.70 | 10.90 | 2.55 | 89.29 | 109.25 | 82.81 | 0.36 |
| 12DS-25 | G2 | 28.52 | 1.51 | 10.44 | 2.61 | 0.52 | 140.15 | 39.26 | 5.98 | 118.40 | 56.00 | 90.00 | 220.01 | 26.20 | 11.40 | 3.11 | 80.75 | 146.60 | 127.45 | 0.37 |
| 75-1-127 | G2 | 28.52 | 1.38 | 11.88 | 2.45 | 0.53 | 163.46 | 38.60 | 5.94 | 128.50 | 40.00 | 73.40 | 166.65 | 23.25 | 11.80 | 2.59 | 80.37 | 132.90 | 73.93 | 0.31 |
| Apo | G2 | 28.68 | 1.28 | 11.76 | 2.46 | 0.55 | 148.32 | 36.86 | 5.96 | 119.60 | 56.00 | 76.70 | 167.46 | 27.35 | 12.65 | 2.78 | 84.12 | 170.85 | 113.81 | 0.41 |
| CT18615 | G2 | 27.60 | 1.15 | 10.85 | 2.59 | 0.51 | 168.10 | 32.95 | 5.13 | 122.50 | 56.00 | 89.50 | 186.66 | 27.50 | 12.35 | 2.80 | 83.95 | 145.15 | 89.41 | 0.32 |
| CT6946 | G2 | 29.06 | 1.21 | 11.92 | 2.46 | 0.51 | 173.45 | 37.44 | 5.97 | 132.80 | 40.00 | 79.90 | 155.76 | 25.98 | 11.95 | 2.33 | 84.13 | 131.35 | 56.36 | 0.27 |
| FED CARE | G2 | 30.20 | 1.33 | 10.70 | 2.86 | 0.51 | 172.08 | 39.38 | 5.55 | 118.50 | 64.00 | 85.40 | 188.25 | 24.60 | 11.50 | 2.46 | 88.01 | 124.15 | 100.72 | 0.35 |
| HHZ 1 | G2 | 28.58 | 1.34 | 11.28 | 2.59 | 0.54 | 132.80 | 37.15 | 5.75 | 117.90 | 45.00 | 79.60 | 141.60 | 23.60 | 12.66 | 2.04 | 95.55 | 99.30 | 59.80 | 0.30 |
| IR07F102 | G2 | 28.66 | 1.12 | 10.55 | 2.75 | 0.50 | 162.97 | 35.56 | 5.60 | 117.00 | 45.00 | 104.10 | 212.17 | 25.30 | 12.55 | 2.65 | 84.97 | 152.90 | 96.95 | 0.31 |
| IR09F436 | G2 | 29.44 | 1.22 | 11.70 | 2.53 | 0.53 | 177.87 | 31.95 | 5.38 | 90.10 | 21.00 | 70.50 | 87.07 | 21.30 | 12.50 | 1.96 | 77.54 | 141.70 | 37.81 | 0.30 |
| IR09L324 | G2 | 30.04 | 0.91 | 10.41 | 2.94 | 0.50 | 155.38 | 39.33 | 6.02 | 91.30 | 76.00 | 69.70 | 149.95 | 18.25 | 11.50 | 2.12 | 68.88 | 138.20 | 104.57 | 0.41 |
| IR09N537 | G2 | 29.02 | 1.22 | 10.89 | 2.81 | 0.54 | 170.20 | 31.45 | 4.76 | 121.60 | 67.00 | 75.10 | 176.89 | 25.35 | 11.70 | 2.17 | 79.21 | 112.05 | 102.63 | 0.37 |
| IR10A134 | G2 | 27.56 | 1.22 | 11.54 | 2.52 | 0.52 | 167.44 | 31.65 | 5.57 | 95.70 | 17.00 | 76.10 | 87.41 | 23.08 | 11.65 | 2.15 | 89.48 | 149.15 | 38.88 | 0.31 |
| IR49830 | G2 | 27.96 | 1.21 | 12.05 | 2.33 | 0.59 | 152.34 | 28.35 | 5.38 | 85.60 | 18.00 | 67.50 | 79.50 | 19.38 | 10.40 | 1.76 | 86.01 | 113.75 | 71.03 | 0.47 |
| R6-PAK | G2 | 30.12 | 1.11 | 11.52 | 2.75 | 0.54 | 160.11 | 36.20 | 6.00 | 108.80 | 33.00 | 84.50 | 131.79 | 24.60 | 12.30 | 3.25 | 78.86 | 177.55 | 66.75 | 0.34 |
| IR78049 | G2 | 27.16 | 1.33 | 12.50 | 2.22 | 0.51 | 172.59 | 39.88 | 6.52 | 129.40 | 34.00 | 77.10 | 128.82 | 23.48 | 14.55 | 2.81 | 79.39 | 133.80 | 49.08 | 0.28 |
| IR85422 | G2 | 27.68 | 1.23 | 11.33 | 2.45 | 0.52 | 159.99 | 37.72 | 5.31 | 98.30 | 33.00 | 73.65 | 98.56 | 23.94 | 10.90 | 2.24 | 75.78 | 127.40 | 55.45 | 0.36 |
| IR86174-2 | G2 | 23.72 | 0.75 | 9.19 | 2.60 | 0.52 | 161.98 | 30.74 | 5.02 | 110.50 | 46.00 | 93.00 | 159.28 | 26.65 | 13.25 | 3.08 | 84.61 | 151.25 | 78.61 | 0.33 |
| IR93323 | G2 | 32.12 | 1.39 | 12.09 | 2.70 | 0.54 | 164.70 | 30.81 | 4.67 | 137.90 | 39.00 | 93.00 | 161.40 | 24.40 | 12.90 | 2.43 | 88.40 | 130.25 | 71.66 | 0.31 |
| PALMAR 18 | G2 | 27.02 | 1.32 | 11.59 | 2.44 | 0.53 | 163.12 | 33.77 | 5.67 | 119.20 | 72.00 | 82.80 | 213.44 | 26.43 | 11.35 | 2.60 | 80.50 | 127.60 | 113.16 | 0.35 |
| Thad | G2 | 30.60 | 1.32 | 12.18 | 2.54 | 0.48 | 156.87 | 36.61 | 6.06 | 100.40 | 19.00 | 70.20 | 100.81 | 24.75 | 13.95 | 3.05 | 66.62 | 147.20 | 47.74 | 0.32 |
| CT18372 | G3 | 29.28 | 0.94 | 10.27 | 2.86 | 0.47 | 154.23 | 33.75 | 5.23 | 140.00 | 58.00 | 70.60 | 171.83 | 22.43 | 13.15 | 1.43 | 54.09 | 103.85 | 43.30 | 0.20 |
| CT18614 | G3 | 24.06 | 0.78 | 9.89 | 2.43 | 0.51 | 160.55 | 41.15 | 6.32 | 136.70 | 64.00 | 77.40 | 191.73 | 27.18 | 13.40 | 2.00 | 65.90 | 117.30 | 65.74 | 0.26 |
| CT19561 | G3 | 30.54 | 0.94 | 11.27 | 2.64 | 0.42 | 137.70 | 29.86 | 4.23 | 127.30 | 55.00 | 69.50 | 136.96 | 23.30 | 9.65 | 1.74 | 82.08 | 95.53 | 54.83 | 0.29 |
| CT6510 | G3 | 29.44 | 1.14 | 11.47 | 2.56 | 0.54 | 147.28 | 30.83 | 4.63 | 154.50 | 72.00 | 71.30 | 227.28 | 17.37 | 10.15 | 0.92 | 48.37 | 83.25 | 35.61 | 0.14 |
| IR05F102 | G3 | 23.82 | 0.59 | 9.31 | 2.62 | 0.59 | 133.53 | 34.83 | 5.47 | 119.80 | 74.00 | 79.10 | 279.45 | 30.13 | 11.00 | 2.47 | 77.76 | 126.15 | 99.93 | 0.26 |
| IR05N412 | G3 | 29.76 | 1.41 | 11.83 | 2.55 | 0.53 | 144.65 | 29.50 | 4.57 | 124.60 | 49.00 | 66.50 | 152.80 | 21.53 | 11.05 | 1.98 | 72.38 | 93.10 | 72.83 | 0.32 |
| IR09L179 | G3 | 20.80 | 0.91 | 9.81 | 2.19 | 0.52 | 133.92 | 30.54 | 4.76 | 94.10 | 13.50 | 60.60 | 65.51 | 19.85 | 10.80 | 1.46 | 79.58 | 105.00 | 29.94 | 0.31 |
| IR85411 | G3 | 27.42 | 1.00 | 10.34 | 2.76 | 0.54 | 157.44 | 29.94 | 4.72 | 104.37 | 85.00 | 71.50 | 256.50 | 24.11 | 12.15 | 2.44 | 78.82 | 139.50 | 73.85 | 0.22 |
| IR85427 | G3 | 22.78 | 0.74 | 9.60 | 2.51 | 0.54 | 141.49 | 30.66 | 5.44 | 140.00 | 56.00 | 112.90 | 308.36 | 26.60 | 11.85 | 2.23 | 55.67 | 163.00 | 58.52 | 0.16 |
| IR86052 | G3 | 27.06 | 0.84 | 10.43 | 2.59 | 0.52 | 158.44 | 37.94 | 6.47 | 114.30 | 49.00 | 85.80 | 186.23 | 23.78 | 12.70 | 1.48 | 73.57 | 93.76 | 70.80 | 0.28 |
| IR86126 | G3 | 29.46 | 0.83 | 11.11 | 2.74 | 0.53 | 159.62 | 34.55 | 5.31 | 117.20 | 58.00 | 101.20 | 244.95 | 25.80 | 11.15 | 2.13 | 88.43 | 92.07 | 67.26 | 0.22 |
| IR86174-3 | G3 | 26.30 | 1.04 | 10.58 | 2.52 | 0.51 | 166.01 | 30.92 | 4.83 | 100.70 | 30.00 | 78.70 | 105.48 | 23.75 | 11.75 | 1.61 | 78.88 | 102.70 | 42.57 | 0.29 |
| IR86635 | G3 | 24.34 | 1.17 | 9.74 | 2.56 | 0.50 | 141.06 | 33.06 | 5.18 | 160.67 | 43.00 | 74.35 | 226.03 | 16.75 | 9.47 | 1.02 | 76.88 | 63.79 | 19.14 | 0.08 |
| IR88633 | G3 | 27.94 | 0.83 | 11.27 | 2.57 | 0.48 | 126.75 | 38.83 | 6.28 | 121.63 | 80.00 | 76.87 | 255.74 | 19.16 | 10.59 | 1.26 | 85.52 | 82.41 | 71.80 | 0.22 |
| IR93324 | G3 | 24.86 | 1.06 | 10.17 | 2.49 | 0.62 | 143.43 | 40.86 | 6.33 | 150.75 | 66.00 | 71.88 | 366.50 | 20.26 | 10.28 | 1.18 | 75.46 | 145.76 | 36.84 | 0.09 |
| MIL 240 | G3 | 26.98 | 0.75 | 10.14 | 2.67 | 0.51 | 138.02 | 36.03 | 5.62 | 109.20 | 46.00 | 83.80 | 156.01 | 26.41 | 11.05 | 1.81 | 79.37 | 118.55 | 65.04 | 0.29 |
| MTU1010 | G3 | 27.50 | 0.75 | 10.24 | 2.72 | 0.46 | 144.62 | 37.08 | 5.62 | 119.00 | 70.00 | 81.37 | 274.33 | 21.22 | 11.35 | 1.64 | 64.71 | 105.95 | 50.11 | 0.15 |
| BR47 | G4 | 34.90 | 1.14 | 12.88 | 2.72 | 0.53 | 143.60 | 47.02 | 7.62 | 136.40 | 70.00 | 85.20 | 260.49 | 24.40 | 12.55 | 1.45 | 59.77 | 99.35 | 38.27 | 0.13 |
| CT18593 | G4 | 29.62 | 0.94 | 10.89 | 2.72 | 0.49 | 150.63 | 37.56 | 5.42 | 156.75 | 53.00 | 49.11 | 182.12 | 24.56 | 12.76 | 1.69 | 29.15 | 100.76 | 20.11 | 0.10 |
| FED 2000 | G4 | 33.88 | 1.46 | 12.48 | 2.74 | 0.54 | 168.40 | 34.41 | 5.01 | 145.70 | 78.00 | 99.10 | 256.44 | 26.35 | 12.50 | 1.65 | 60.77 | 112.55 | 57.55 | 0.18 |
| IR04A115 | G4 | 30.68 | 1.13 | 11.26 | 2.80 | 0.52 | 143.10 | 31.85 | 4.78 | 141.40 | 100.50 | 75.70 | 268.22 | 25.95 | 13.05 | 1.41 | 59.13 | 118.60 | 49.92 | 0.16 |
| IR06N155 | G4 | 29.32 | 0.89 | 10.26 | 2.67 | 0.56 | 188.53 | 37.87 | 5.66 | 147.70 | 93.00 | 76.80 | 362.30 | 23.75 | 10.15 | 0.69 | 53.08 | 98.90 | 29.86 | 0.08 |
| IR65482 | G4 | 35.32 | 1.51 | 12.26 | 2.93 | 0.50 | 151.89 | 47.76 | 7.54 | 139.50 | 47.00 | 83.80 | 203.04 | 22.97 | 11.35 | 1.94 | 52.88 | 116.24 | 42.00 | 0.17 |
| IR65600 | G4 | 35.04 | 1.77 | 12.31 | 2.92 | 0.55 | 154.65 | 34.36 | 5.00 | 150.20 | 40.00 | 72.30 | 172.92 | 17.05 | 8.09 | 0.75 | 25.16 | 60.12 | 18.80 | 0.10 |
| IR70213 | G4 | 30.46 | 1.25 | 12.08 | 2.53 | 0.45 | 142.95 | 41.42 | 6.91 | 149.80 | 70.00 | 81.50 | 278.27 | 22.31 | 10.40 | 1.43 | 35.81 | 85.44 | 30.18 | 0.10 |
| IR74371 | G4 | 35.16 | 1.31 | 12.20 | 2.91 | 0.52 | 143.93 | 49.04 | 7.71 | 141.90 | 63.00 | 71.60 | 225.09 | 23.73 | 10.90 | 0.98 | 18.38 | 97.97 | 22.86 | 0.09 |
| IR75483 | G4 | 31.98 | 1.47 | 12.66 | 2.53 | 0.48 | 179.43 | 40.62 | 6.38 | 146.80 | 58.00 | 80.70 | 255.47 | 23.72 | 11.34 | 1.65 | 36.81 | 112.34 | 38.63 | 0.13 |
| IRRI 123 | G4 | 29.94 | 1.50 | 12.10 | 2.53 | 0.54 | 159.81 | 35.36 | 5.57 | 146.98 | 64.00 | 65.81 | 217.34 | 23.36 | 9.98 | 1.13 | 73.75 | 145.17 | 59.49 | 0.22 |
| IRRI 152 | G4 | 26.52 | 0.93 | 9.20 | 2.95 | 0.53 | 181.71 | 38.16 | 5.83 | 130.78 | 70.00 | 114.77 | 368.36 | 24.13 | 16.15 | 0.67 | 66.50 | 100.52 | 35.70 | 0.09 |
| IRRI 154 | G4 | 28.46 | 1.13 | 10.83 | 2.62 | 0.52 | 178.03 | 45.33 | 6.54 | 131.70 | 71.00 | 73.50 | 197.32 | 23.10 | 11.75 | 1.80 | 75.75 | 99.55 | 72.50 | 0.27 |
| IRRI 157 | G4 | 25.40 | 0.85 | 9.89 | 2.76 | 0.52 | 154.75 | 45.64 | 7.12 | 118.88 | 79.00 | 81.50 | 377.85 | 19.99 | 10.80 | 0.94 | 51.91 | 98.23 | 50.59 | 0.12 |
| WAB 56-125 | G4 | 33.08 | 1.33 | 12.24 | 2.71 | 0.51 | 167.97 | 40.85 | 5.88 | 145.00 | 48.00 | 93.50 | 201.18 | 25.18 | 12.35 | 1.25 | 57.85 | 82.70 | 27.50 | 0.12 |
